# Prevalence of comorbid pathological gambling in substance use disorders

**DOI:** 10.1192/j.eurpsy.2023.1190

**Published:** 2023-07-19

**Authors:** P. Cargonja, S. Jonovska, V. Šendula Jengić, T. Sugnet

**Affiliations:** 1 Department of Addiction and Psychotrauma; 2 County Hospital Insula, Rab, Croatia

## Abstract

**Introduction:**

Since gambling opportunities expanded over the last four decades, gambling, including pathological and problem gambling, has received increased attention from clinicians and researchers worldwide.

**Objectives:**

Prevalence of gambling disorders varies according to the screening instruments, measurement used as well as accessibility of gambling opportunities but it is believed that gambling disorders affect 0.2–5.3% of adults worldwide. In addition, considering that the gambling disorders are highly comorbid with other substance use and mental health disorders, for both the causes and treatment implications of this disorder a further understanding is needed.

**Methods:**

This research has been conducted at the Addiction and Psychotrauma Department of the Insula County Hospital over a period of two months on a sample of 150 people using a questionnaire that was distributed to patients whose primary diagnosis was substance use disorder but did not have a diagnosed gambling addiction with the aim of early detection of it.

**Results:**

Substance abuse may include minimizing one’s use, hiding other comorbid addictions including gambling, and an underestimation of the effect one’s use has on life areas as well as family members.

**Conclusions:**

This article highlights the prevalence of comorbid unrecognised pathological gambling in substance use disorders, but also reviews definition, clinical similarities and differences and treatment approaches.

**Disclosure of Interest:**

None Declared

